# Retrospective Electronic Medical Records Study to Understand the Usage Patterns and Patient Profiles of Those Prescribed Amlodipine and Its Combinations

**DOI:** 10.7759/cureus.88954

**Published:** 2025-07-29

**Authors:** Sunil Lhila, Arindam Pande, Rohit Kumar, Swathi Bhureddy, Arti Sanghavi, Sagar Katare, Jay Shah, Snehal Shah, Garima Verma

**Affiliations:** 1 Cardiology, Rabindranath Tagore International Institute of Cardiac Sciences, Kolkata, IND; 2 Cardiology, Medica Superspecialty Hospital, Kolkata, IND; 3 Medical Affairs, Dr. Reddy's Laboratories Ltd., Hyderabad, IND; 4 Medical Affairs, Dr. Reddy's Laboratories, Hyderabad, IND; 5 Medical Affairs, Dr. Reddy’s Laboratories, Hyderabad, IND; 6 Cardiology, HCG Multispeciality Hospital, Ahmedabad, IND; 7 Clinical Insights, HealthPlix Technologies Private Limited, Bengaluru, IND

**Keywords:** amlodipine, calcium channel blocker, electronic medical records, hypertension, pedal edema

## Abstract

Background: Hypertension is a lifestyle disorder with a target blood pressure (BP) <140/90 mmHg. Amlodipine, a calcium channel blocker (CCB), is the first-line choice of treatment. This study aims to assess the effectiveness, tolerability, and treatment pattern of amlodipine or its combinations in patients with hypertension.

Methodology: This retrospective, electronic medical records (EMR)-based longitudinal study analyzed anonymized data of hypertensive patients with prescriptions for amlodipine or its combinations. The primary aim was to understand the usage patterns, while the secondary goals were to assess effectiveness and tolerability. Outcome measures included changes in mean systolic BP (SBP) and diastolic BP (DBP), and the incidence of pedal edema.

Results: Most of the participants were aged 40 to 64 years (65.47%) and were predominantly male (52.85%). Dyslipidemia and diabetes mellitus (DM) were the most common comorbidities. There was a statistically significant reduction in SBP and DBP from visit 1 to visit 2 with amlodipine or its combination therapies (p < 0.001), irrespective of any comorbid condition. Pedal edema occurred in 0.91% of patients, notably lower than the reported 1.7%-32%.

Conclusion: Amlodipine or its combinations demonstrated a significant reduction in BP with a very low incidence of pedal edema. Hence, amlodipine shows good tolerability and effectiveness in the Indian population.

## Introduction

The European Society of Hypertension (ESH) 2018 guidelines define hypertension as the level of blood pressure (BP) at which the benefits of treatment (either with lifestyle interventions or drugs) unequivocally outweigh the risks of treatment, as documented by clinical trials [1]. The 2023 ESH guidelines recommend a threshold of >140/90 mmHg (grade 1) for diagnosing hypertension, whereas the American Heart Association (AHA) recommends a lower threshold of >130/80 mmHg (stage 1) [2].

The National Family Health Survey (NFHS)-5 reports the prevalence of hypertension in India to be 18.3% [3]. A recent systematic review reported that, during 2001-2022, only 22.5% of hypertensive patients in India had controlled BP [4]. Despite the rising prevalence and negative consequences of hypertension, awareness and control of hypertension remain poor in India. Long-term studies indicate that hypertensive patients need an average of three drugs for effective BP control, rather than monotherapy [5].

The India Hypertensive Control Initiative (IHCI) recommends amlodipine as the first-line treatment of hypertension, followed by telmisartan and a diuretic [6]. Amlodipine, a third-generation dihydropyridine calcium channel blocker (CCB), lowers peripheral vascular resistance by vasodilating smooth muscle cells. Its high oral bioavailability (60-80%), long half-life (35-50 hours), and lipophilic nature enhance patient compliance [7]. Amlodipine is primarily indicated to treat angina and hypertension [7]. Peripheral edema can occur with amlodipine use; however, administering it at bedtime and in lower doses (2.5 or 5 mg/day) can mitigate this side effect [8]. A review found that amlodipine had a neutral effect across various comorbid conditions in hypertension trials, whether used alone or with diuretics, angiotensin-converting enzyme inhibitors (ACEi), or angiotensin receptor blockers (ARB) [9]. This electronic medical records (EMR) study was planned to understand the usage, effectiveness, and tolerability of amlodipine and its combinations.

## Materials and methods

Study design

This was a retrospective, EMR-based study that used anonymized data for the period January 2020 to May 2023. Patients diagnosed with primary hypertension of either gender, aged ≥18 years, prescribed amlodipine or its combinations with or without other antihypertensive agents, and who had a follow-up visit within 10-30 days of the baseline visit were included. Data from patients with hypertension due to secondary causes, such as pregnancy-induced hypertension, and those with incomplete data were excluded.

At the baseline visit (visit 1), the patient was prescribed amlodipine or its combinations with or without other antihypertensive agents. Data for age, gender, body mass index (BMI), comorbidities, systolic BP (SBP), and diastolic BP (DBP) were captured.

At the follow-up visit (10-30 days from baseline), the SBP and DBP values recorded were used to evaluate the change in BP. The complaints section of the EMR was reviewed to identify any adverse events.

The primary outcome measure was to understand the usage of amlodipine or its combinations, while the secondary outcomes were to assess effectiveness and tolerability.

The data were collected using a data collection form. Ethics committee (EC) approval was obtained from the Royal Pune Independent Ethics Committee on August 28, 2023 (IEC no.: RPIEC280823), and the study was registered on the Clinical Trials Registry of India (CTRI/2023/09/057949).

Statistical analysis

Continuous variables are summarized using descriptive statistics (sample size (n), mean, standard deviation (SD)). Categorical variables are presented as the number and percentage of patients. Unless otherwise mentioned, percentages were based on the number of patients from the population, as appropriate. All statistical tests were conducted at a two-sided 5% significance level. For paired continuous data, a paired t-test was used.

## Results

**Figure 1:**
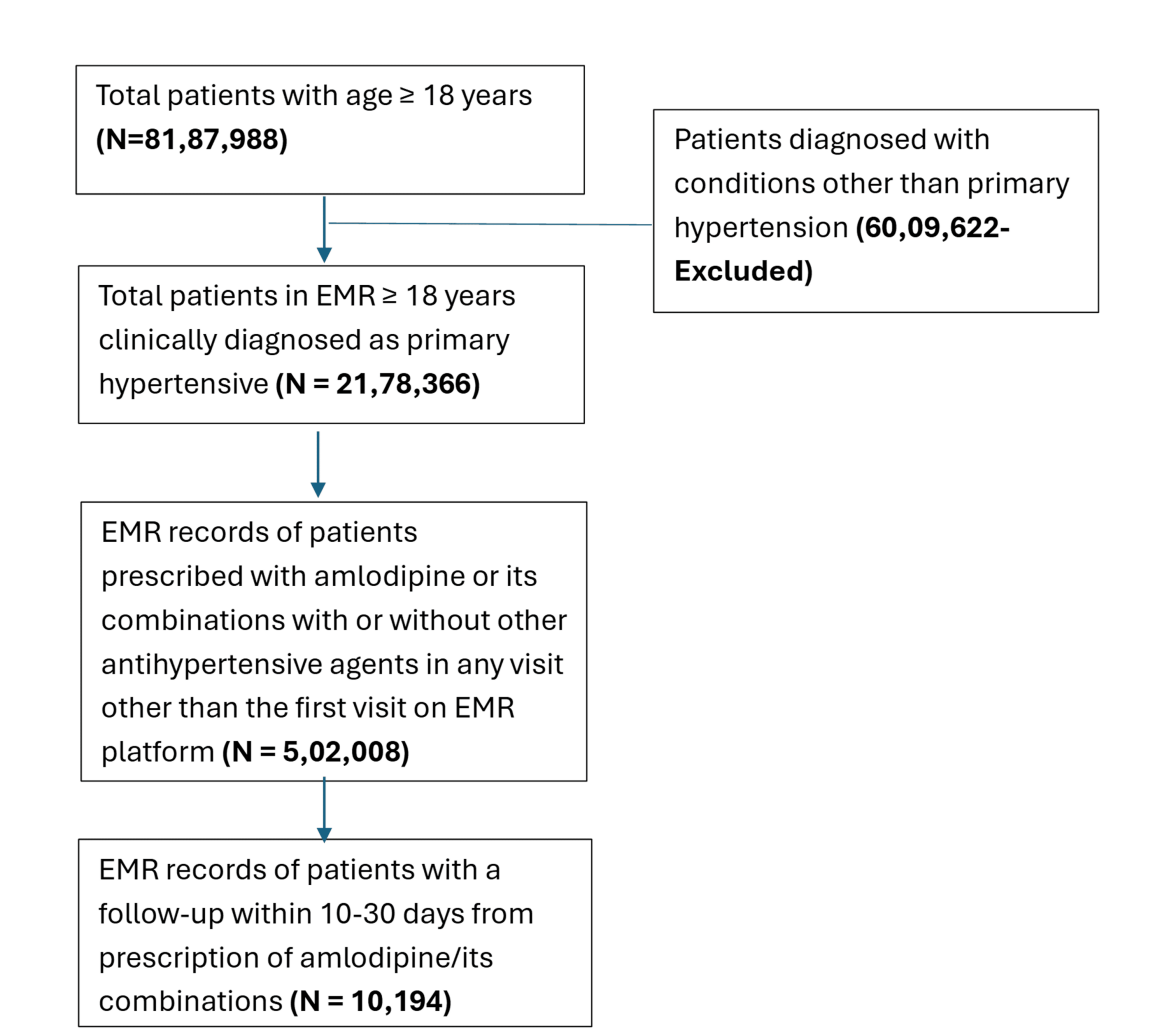
CONSORT diagram CONSORT: Consolidated Standards of Reporting Trials.

From a total of 9,024,599 records in the EMR from 2020 to 2023, 2,178,366 adult (≥18 years) patients (26.6%) were hypertensive. Of these, 502,008 (23.0%) were prescribed amlodipine or its combinations, while only 10,194 patients (2.0%) had a follow-up within 10-30 days (Figure 1).

Baseline characteristics of patients prescribed amlodipine or its combinations

Of the 10,194 patients meeting the eligibility criteria, 6,675 (65.47%) were aged 40-64 years, and 2,431 (23.84%) were ≥65 years. The majority were male (n = 5,388, 52.85%). Dyslipidemia, DM, and dyslipidemia + DM were the most common comorbidities. Other comorbidities included CKD, CAD, dyslipidemia with CKD, dyslipidemia with CAD, DM with CKD, DM with CAD, CAD with CKD, dyslipidemia with DM and CKD, dyslipidemia with DM and CAD, dyslipidemia with CAD and CKD, DM with CAD and CKD, and dyslipidemia with DM, CAD, and CKD, each accounting for ≤10% of patients in the individual group (Table 1).

**Table 1:** Summary of demographic characteristics

Parameters		Overall	Amlodipine (n=2460)	Amlodipine+ telmisartan (n=2847)	Amlodipine+ atenolol (n=477)	Amlodipine+ bisoprolol (n=639)	Amlodipine+ metoprolol (n=1356)	Amlodipine+ chlorthalidone+ telmisartan (n=429)	Amlodipine+ hydrochlorothiazide+ telmisartan (n=1557)	Amlodipine+ hydrochlorothiazide+ olmesartan (n=429)
Age, N (mean ±SD)	18-39	1,088 (33.91±4.45)	182 (32.9±4.9)	264 (34.5±4.2)	44 (34.6±3.9)	91 (34.4±3.8)	174 (33.5±4.6)	28 (34.7±3.1)	145 (34.6±3.8)	36 (34.2±5.0)
	40-64	6,675 (52.96±6.86)	1513 (52.9±6.9)	1882 (53.0±6.8)	316 (53.2±6.8)	415 (52.8±6.8)	894 (52.8±7.0)	298 (52.9±6.9)	1079 (53.1±6.9)	278 (53.4±6.5)
	≥ 65	2,431 (70.51±5.21)	641 (70.9±5.5)	701 (70.4±5.1)	117 (70.6±5.7)	133 (70.8±5.4)	288 (70.6±4.8)	103 (71.6±5.7)	333 (69.8±4.7)	115 (69.8±5.3)
Gender, n (%)	Male	5,388 (52.85)	1334 (54.2)	1505 (52.9)	241 (50.5)	345 (54.0)	692 (51.0)	233 (54.3)	816 (52.4)	222 (51.7)
Comorbities, n (%)	Dyslipidemia	-	878 (35.69%)	1051 (36.92%)	160 (33.54%)	285 (44.60%)	550 (40.56%)	166 (38.69%)	572 (36.74%)	179 (41.72%)
	DM	-	816 (33.17%)	1306 (45.87%)	199 (41.72%)	222 (34.74%)	472 (34.81%)	183 (42.66%)	691 (44.38%)	197 (45.92%)
	CKD	-	243 (9.88%)	65 (2.28%)	27 (5.66%)	34 (5.32%)	98 (7.23%)	8 (1.86%)	14 (0.90%)	7 (1.63%)
	CAD	-	185 (7.52%)	165 (5.80%)	39 (8.18%)	68 (10.64%)	104 (7.67%)	25 (5.83%)	87 (5.59%)	30 (6.99%)
	Dyslipidemia+DM	-	419 (17.03%)	601 (21.11%)	87 (18.24%)	132 (20.66%)	265 (19.54%)	81 (18.88%)	320 (20.55%)	103 (24.01%)
	Dyslipidemia+CKD	-	85 (3.46%)	33 (1.16%)	15 (3.14%)	23 (3.60%)	45 (3.32%)	3 (0.70%)	10 (0.64%)	4 (0.93%)
	Dyslipidemia+CAD	-	150 (6.10%)	120 (4.21%)	30 (6.29%)	54 (8.45%)	88 (6.49%)	18 (4.20%)	69 (4.43%)	22 (5.13%)
	DM+CKD	-	72 (2.93%)	30 (1.05%)	9 (1.89%)	17 (2.66%)	44 (3.24%)	7 (1.63%)	12 (0.77%)	3 (0.70%)
	DM+CAD	-	79 (3.21%)	75 (2.63%)	18 (3.77%)	24 (3.76%)	47 (3.47%)	9 (2.10%)	33 (2.12%)	13 (3.03%)
	CAD+CKD	-	22 (0.89%)	7 (0.25%)	2 (0.42%)	7 (1.10%)	10 (0.74%)	1 (0.23%)	3 (0.19%)	1 (0.23%)
	Dyslipidemia+DM+CKD	-	38 (1.54%)	18 (0.63%)	8 (1.68%)	13 (2.03%)	27 (1.99%)	3 (0.70%)	8 (0.51%)	2 (0.47%)
	Dyslipidemia+DM+CAD	-	66 (2.68%)	57 (2.00%)	16 (3.35%)	21 (3.29%)	43 (3.17%)	7 (1.63%)	29 (1.86%)	11 (2.56%)
	Dyslipidemia+CAD+CKD		17 (0.69%)	5 (0.18%)	2 (0.42%)	5 (0.78%)	8 (0.59%)		2 (0.13%)	1 (0.23%)
	DM+CAD+CKD		9 (0.37%)	5 (0.18%)	1 (0.21%)	3 (0.47%)	7 (0.52%)	1 (0.23%)	3 (0.19%)	
	Dyslipidemia + DM + CAD + CKD		8 (0.33%)	3 (0.11%)	1 (0.21%)	3 (0.47%)	6 (0.44%)		2 (0.13%)	

Change in SBP and DBP from baseline to follow-up

There was a statistically significant reduction in SBP and DBP from visit 1 to visit 2 with amlodipine or its combination therapies (p < 0.001), irrespective of comorbid condition (Appendices).

In patients prescribed amlodipine, the mean ± SD reduction in SBP was 17.8 ± 19.6 mmHg, and in DBP was 9.5 ± 10.7 mmHg.

In patients prescribed amlodipine + telmisartan, the mean ± SD reduction in SBP was 21.6 ± 19.2 mmHg. In patients prescribed amlodipine and a β-blocker, the maximum mean reduction in SBP/DBP was observed with amlodipine + bisoprolol (23.6/12.8 mmHg), followed by amlodipine + atenolol (21.3/11.7 mmHg).

In the case of triple therapy, the maximum mean ± SD reduction in SBP was 25.2 ± 20.9 mmHg with amlodipine + chlorthalidone + telmisartan, followed by 24.9 ± 20.5 mmHg with amlodipine + hydrochlorothiazide + olmesartan. The highest mean ± SD reduction in DBP was 12.9 ± 11.1 mmHg with amlodipine + hydrochlorothiazide + olmesartan, followed by 12.7 ± 10.9 mmHg with amlodipine + chlorthalidone + telmisartan (Table 2).

**Table 2:** Change in SBP and DBP from baseline to follow-up DBP: diastolic blood pressure, SBP: systolic blood pressure, SD: standard deviation. *Paired t-test was used to test if there is a significant change in means from baseline to follow-up visits.

Parameters	SBP, mean ± SD					DBP, mean ± SD				
	Visit 1	Visit 2	Change	T-value	P-value*	Visit 1	Visit 2	Change	T-value	P-value*
Amlodipine (n=2460)	158.6 ± 17.3	140.8 ± 16.2	-17.8 ± 19.6	45.15	<0.001	95.0 ± 8.4	85.4 ± 9.2	-9.5 ± 10.7	44.37	<0.001
Amlodipine+ telmisartan (n=2847)	161.0 ± 16.9	139.4 ± 16.6	-21.6 ± 19.2	60.25	<0.001	95.8 ± 8.3	84.4 ± 9.0	-11.4 ± 10.2	59.55	<0.001
Amlodipine+ atenolol (n=477)	159.5 ± 18.1	138.2 ± 17.2	-21.3 ± 21.1	22.1	<0.001	95.3 ± 8.7	83.6 ± 9.4	-11.7 ± 11.5	22.36	<0.001
Amlodipine+ bisoprolol (n=639)	161.6 ± 17.8	137.9 ± 16.1	-23.6 ± 19.0	31.4	<0.001	96.3 ± 8.7	83.4 ± 9.3	-12.8 ± 10.5	31.04	<0.001
Amlodipine+ metoprolol (n=1356)	160.6 ± 17.1	140.7 ± 16.8	-19.8 ± 19.7	37.14	<0.001	95.9 ± 8.7	85.5 ± 9.3	-10.4 ± 10.6	35.84	<0.001
Amlodipine+ chlorthalidone+ telmisartan (n=429)	163.0 ± 17.3	137.8 ± 16.0	-25.2 ± 20.9	24.92	<0.001	96.0 ± 8.6	83.2 ± 8.9	-12.7 ± 10.9	24.22	<0.001
Amlodipine+ hydrochlorothiazide+ telmisartan (n=1557)	163.2 ± 17.2	139.7 ± 16.8	-23.4 ± 19.9	46.42	<0.001	96.6 ± 9.1	84.6 ± 9.2	-12.0 ± 10.6	44.48	<0.001
Amlodipine+ hydrochlorothiazide+ olmesartan (n=429)	166.3 ± 17.4	141.4 ± 17.8	-24.9 ± 20.5	25.2	<0.001	98.0 ± 8.9	85.1 ± 9.4	-12.9 ± 11.1	24.23	<0.001

Proportion of patients with pedal edema at follow-up

Patients who presented with complaints of pedal edema at baseline were excluded from this analysis. Of the 10,106 patients considered for this analysis, only 31 (1.28%) of 2,430 patients on amlodipine reported pedal edema. Among patients on amlodipine dual therapy, 20 (0.71%) of 2,835 patients on amlodipine + telmisartan and 27 (1.10%) of 2,454 patients on amlodipine + β-blocker reported pedal edema. Of the 2,387 patients prescribed triple therapy (amlodipine + diuretic + ARB), 14 (5.86%) complained of pedal edema at follow-up (Table 3).

**Table 3:** Proportion of patients with pedal edema at follow-up Patients with pedal edema (n=88) at baseline visit are excluded from the analysis of the incidence of pedal edema. CI: confidence interval.

Parameter	Category	Overall (N = 10,106), n (%)	95% CI for the proportion
Patients with pedal edema	Amlodipine (n=2,430)	31 (1.28%)	(0.83; 1.72)
	Amlodipine+Atenolol (n=476)	8 (1.68%)	(0.53; 2.84)
	Amlodipine+Bisoprolol (n=638)	4 (0.63%)	(0.01; 1.24)
	Amlodipine+Chlorthalidone+Telmisartan (n=424)	3 (0.71%)	(-0.09; 1.51)
	Amlodipine+Hydrochlorothiazide+Olmesartan (n=425)	3 (0.71%)	(-0.09; 1.5)
	Amlodipine+Hydrochlorothiazide+Telmisartan (n=1538)	8 (0.52%)	(0.16; 0.88)
	Amlodipine+Metoprolol (n=1340)	15 (1.12%)	(0.56; 1.68)
	Amlodipine+Telmisartan (n=2835)	20 (0.71%)	(0.4; 1.01)

## Discussion

In this study, most hypertensive patients were >40 years, indicating a potential association between the prevalence of hypertension and increasing age [10]. More than 70% of patients were overweight and obese, presenting obesity as one of the major risk factors for hypertension [11]. The most common comorbidities were dyslipidemia and DM, consistent with existing literature [12].

A recent study published by IHCI concluded that simple treatment plans can effectively control BP in a large-scale real-world scenario. It recommends starting with one low-cost medication and, if necessary, adding or switching to another antihypertensive based on availability [13]. In alignment with this, our results highlight that amlodipine and its combinations were effective in controlling BP.

Amlodipine monotherapy significantly reduced SBP and DBP. A meta-analysis that included 85 studies reported a 12 mmHg decrease in SBP with amlodipine compared to placebo. Additionally, patients with DM experienced a mean reduction of SBP of 19.1 mmHg, closely aligning with the current study’s finding of 17.8 mmHg reduction [14]. Randomized, double-blind, placebo-controlled studies have demonstrated the efficacy of amlodipine as monotherapy and in combination with other antihypertensive agents such as ARBs, diuretics, and ACEi. These studies suggested that amlodipine is a safe and effective first-line antihypertensive agent [7,15].

A real-world evidence (RWE) study on 24-hour ambulatory blood pressure (ABP) control reported that factors such as daily amlodipine regimen, gender, hypertension severity, and therapy duration did not significantly impact ABP changes in patients receiving either amlodipine monotherapy or combination therapy for essential hypertension. This showed that, irrespective of these factors, amlodipine is effective in all patients [7]. Evidence suggests that increasing the dose of monotherapy decreases coronary events and cerebrovascular events by 29% and 40%, whereas combining antihypertensive drugs with different mechanisms reduces coronary events and cerebrovascular events by 40% and 54%, respectively [5]. Various guidelines have recommended multidrug treatment for managing hypertension, which has led to the incorporation of FDCs. These combinations offer several advantages, including enhanced efficacy, better adherence, and cost-effectiveness [16]. The ACCOMPLISH trial demonstrated these advantages, where more than 70% of patients on FDC achieved BP <140/90 mmHg [17].

Amlodipine + bisoprolol reduced SBP at visit 2 by 23.6 mmHg. This result is supported by a randomized, multicenter, comparative clinical trial where patients who failed on monotherapy with amlodipine or bisoprolol were administered amlodipine + bisoprolol, leading to a reduction in SBP by 25.3 mmHg [18]. The mean change in SBP/DBP in this study with amlodipine + metoprolol was 19.9/10.4 mmHg. Almost similar results were observed in a study where SBP/DBP reduction was 22.88/13.98 mmHg with amlodipine + metoprolol [19]. In our study, 46.47% of patients on dual therapy were given a β-blocker, though IHCI recommends it only for those with heart attack.

With amlodipine + chlorthalidone + telmisartan, a reduction in SBP of 25.2 mmHg at visit 2 was observed in this study. This is comparable to a phase 3 trial where patients on amlodipine + chlorthalidone + telmisartan showed a 19.1 mmHg reduction in SBP after 8 weeks of treatment [20]. The mean change in SBP/DBP in this study with amlodipine + hydrochlorothiazide + olmesartan was −24.9/−12.9 mmHg at visit 2. Similar mean changes of −17.8/−9.3 mmHg within 4 weeks were observed in a real-world study in patients treated with amlodipine + hydrochlorothiazide + olmesartan [21]. Additionally, amlodipine + hydrochlorothiazide + telmisartan reduced SBP by 23.5 mmHg, similar to an 18.7 mmHg reduction observed in another phase 3 trial [22].

Amlodipine is generally associated with vasodilatory effects and pedal edema, which may lead to its discontinuation. Since this is a dose-dependent effect, low-dose combinations or combinations with ARBs or diuretics can reduce edema, as ARBs can counteract the circulatory changes that cause edema [23]. Although the literature suggests an incidence rate between 1.7% and 32% for pedal edema with CCBs and their combinations, this study found a lower incidence of 0.91% [24,25]. This is supported by another EMR-based RWE study, which showed better tolerability for amlodipine with fewer cases of pedal edema [26]. The lower incidence observed in this study could also be attributed to the action of diuretics.

Amlodipine effectively manages hypertension while reducing major cardiovascular events such as hospitalizations, atherosclerotic changes, transient ischemic attacks, strokes, and non-fatal myocardial infarctions [17]. Unlike other CCBs, amlodipine enhances nitric oxide (NO) production in heart failure. Its antioxidant and anti-inflammatory effects confer a vasoprotective benefit [8]. The IHCI protocol recommends starting amlodipine at 5 mg and increasing to 10 mg if BP remains uncontrolled [6]. In addition to its efficacy in enhancing cardiovascular health and reducing major cardiovascular outcomes, amlodipine is also a cost-effective choice for managing hypertension and is more affordable than many other antihypertensive agents [27,28].

A few limitations of this retrospective real-world study include inconsistent follow-up durations, the presence of missing data, and limited control over patient characteristics and other variables. The absence of a comparison group limits attribution of effect size solely to the intervention. Furthermore, BP reduction was the only primary endpoint considered; clinical outcomes such as cardiovascular events were not assessed.

## Conclusions

This study concludes that amlodipine and its combination therapies were associated with a marked reduction in SBP and DBP in patients with hypertension, including those with comorbid conditions such as diabetes mellitus, dyslipidemia, CKD, and CAD. Combination therapies, particularly triple therapy of amlodipine, a diuretic, and an ARB, were associated with the greatest BP reduction, suggesting that the antihypertensive effects are enhanced when agents with complementary mechanisms of action are used. The overall incidence of pedal edema was low, particularly with combination therapies. Amlodipine, being one of the most affordable antihypertensives available, could be considered as an option in various non-communicable disease programs; however, this warrants further validation through controlled trials and cost-benefit analysis studies. Further studies with control over demographics and other variables would be helpful to strengthen the current observations.

## Appendices

**Table 4:** Change in SBP and DBP from baseline to follow-up by comorbidities Supplementary Table 1 Comb. 1: Amlodipine+Telmisartan, Comb. 2: Amlodipine+Atenolol, Comb. 3: Amlodipine+Bisoprolol, Comb. 4: Amlodipine+Metoprolol, Comb. 5: Amlodipine+Chlorthalidone+Telmisartan,Comb. 6: Amlodipine+Hydrochlorothiazide+Telmisartan, Comb. 7: Amlodipine+hydrochlorothiazide+Olmesartan. SBP: systolic blood pressure, DBP: diastolic blood pressure, DM: diabetes mellitus, CAD: coronary artery disease, CKD: chronic kidney disease.

Comorbidity	Visit	Statistics (N=10,194)	Amlodipine	Comb. 1	Comb. 2	Comb. 3	Comb. 4	Comb. 5	Comb. 6	Comb. 7
Dyslipidemia	Visit 1 SBP	n	878	1051	160	285	550	166	572	179
		Mean(SD)	158.4(17.3)	159.8(16.5)	158.9(18.8)	160.8(18.3)	159.7(17.2)	162.3(16.2)	162.6(16.1)	166.3(17.4)
		Median	160.0	160.0	160.0	160.0	160.0	160.0	160.0	165.0
		Min, Max	122,200	121,200	125,200	124,200	121,200	121,200	121,200	121,200
	Visit 2 SBP	Mean(SD)	140.7(15.8)	138.3(15.9)	139.0(17.8)	139.0(16.9)	140.4(17.0)	139.6(16.7)	139.3(17.0)	142.8(17.5)
		Median	140.0	140.0	140.0	140.0	140.0	140.0	140.0	140.0
		Min, Max	100,194	70,200	100,180	101,200	90,200	93,190	94,200	108,200
	Change from Baseline (SBP)	Mean(SD)	-17.8(19.6)	-21.6(18.5)	-19.9(21.4)	-21.8(19.3)	-19.3(20.6)	-22.7(21.5)	-23.3(19.4)	-23.5(21.0)
		Median	-18.0	-20.0	-20.0	-20.0	-20.0	-23.0	-20.0	-20.0
		P value	<0.001	<0.001	<0.001	<0.001	<0.001	<0.001	<0.001	<0.001
Dyslipidemia	Visit 1 DBP	n	878	1051	160	285	550	166	572	179
		Mean(SD)	93.9(7.7)	94.8(8.4)	94.8(8.4)	94.9(8.0)	94.3(7.9)	94.9(8.2)	95.6(8.9)	97.4(9.0)
		Median	90.0	90.0	90.0	90.0	90.0	90.0	93.0	98.0
		Min, Max	81,130	81,150	81,128	81,120	81,140	81,130	81,150	81,120
	Visit 2 DBP	Mean(SD)	84.3(8.8)	83.3(8.9)	83.8(9.1)	83.3(9.4)	84.4(8.9)	83.4(8.8)	83.6(9.1)	85.1(9.7)
		Median	82.5	80.0	80.0	80.0	83.5	80.0	80.0	81.0
		Min, Max	60,140	60,140	60,106	60,120	60,140	62,120	60,130	60,120
	Change from Baseline (DBP)	Mean(SD)	-9.6(10.3)	-11.4(10.3)	-11.0(11.2)	-11.7(10.3)	-9.9(10.3)	-11.5(10.2)	-12.0(10.4)	-12.3(10.9)
		Median	-10.0	-10.0	-10.0	-10.0	-10.0	-10.0	-10.0	-10.0
		P value	<0.001	<0.001	<0.001	<0.001	<0.001	<0.001	<0.001	<0.001
DM	Visit 1 SBP	n	816	1306	199	222	472	183	691	197
		Mean(SD)	159.6(17.6)	160.5(16.6)	159.7(18.5)	162.8(17.8)	161.2(17.4)	162.3(16.8)	162.9(17.3)	167.3(17.6)
		Median	160.0	160.0	160.0	160.0	160.0	160.0	160.0	170.0
		Min, Max	122,200	121,200	122,200	124,200	121,200	126,200	121,200	121,200
	Visit 2 SBP	Mean(SD)	141.5(17.2)	140.5(16.8)	141.4(16.0)	140.7(17.6)	142.6(17.1)	139.3(15.7)	140.7(17.0)	142.6(18.2)
		Median	140.0	140.0	140.0	140.0	140.0	140.0	140.0	140.0
		Min, Max	94,200	70,200	100,184	101,200	90,200	107,180	96,200	100,200
	Change from Baseline (SBP)	Mean(SD)	-18.1(20.0)	-20.0(19.2)	-18.4(20.5)	-22.1(19.4)	-18.6(20.9)	-23.0(21.1)	-22.1(19.8)	-24.6(20.1)
		Median	-20.0	-20.0	-20.0	-20.0	-20.0	-20.0	-20.0	-24.0
		P value	<0.001	<0.001	<0.001	<0.001	<0.001	<0.001	<0.001	<0.001
DM	Visit 1 DBP	Mean(SD)	93.8(7.9)	94.9(7.9)	94.0(8.2)	94.3(6.9)	94.4(8.2)	94.9(7.7)	95.2(8.4)	96.9(8.3)
		Median	90.0	92.0	90.0	90.0	90.0	90.0	92.0	98.0
		Min, Max	81,137	81,150	81,128	82,120	81,140	82,120	81,138	81,120
	Visit 2 DBP	Mean(SD)	84.3(8.8)	83.9(8.7)	83.4(9.1)	83.2(9.5)	84.7(9.2)	82.9(8.7)	84.2(8.9)	85.4(9.1)
		Median	83.0	80.0	80.0	80.0	84.0	80.0	82.0	84.0
		Min, Max	60,116	60,140	60,110	60,115	60,130	60,120	60,122	60,116
	Change from Baseline (DBP)	n	816	1306	199	222	472	183	691	197
		Mean(SD)	-9.4(10.1)	-11.0(10.0)	-10.6(10.5)	-11.1(10.2)	-9.6(10.5)	-12.0(9.1)	-11.1(10.6)	-11.5(10.9)
		Median	-10.0	-10.0	-10.0	-10.0	-10.0	-10.0	-10.0	-10.0
		P value	<0.001	<0.001	<0.001	<0.001	<0.001	<0.001	<0.001	<0.001
CAD	Visit 1 SBP	n	185	165	39	68	104	25	87	30
		Mean(SD)	161.3(17.9)	162.4(16.2)	160.8(21.6)	163.4(19.1)	163.0(15.2)	164.9(10.2)	164.7(15.8)	168.7(19.0)
		Median	160.0	160.0	160.0	161.0	160.0	170.0	165.0	170.0
		Min, Max	122,200	126,200	126,200	130,200	128,200	147,180	126,200	126,200
	Visit 2 SBP	Mean(SD)	142.5(17.1)	139.6(15.7)	138.4(20.5)	139.9(17.3)	141.1(17.8)	139.6(16.3)	139.5(17.2)	145.9(15.3)
		Median	140.0	140.0	140.0	140.0	140.0	140.0	140.0	141.5
		Min, Max	94,194	110,190	100,180	90,180	100,200	113,180	96,190	120,190
	Change from Baseline (SBP)	Mean(SD)	-18.8(21.7)	-22.8(19.9)	-22.4(26.2)	-23.5(21.0)	-21.9(20.7)	-25.3(17.8)	-25.2(21.6)	-22.8(23.8)
		Median	-18.0	-20.0	-20.0	-22.5	-20.0	-20.0	-24.0	-20.0
		P value	<0.001	<0.001	<0.001	<0.001	<0.001	<0.001	<0.001	<0.001
CAD	Visit 1 DBP	n	185	165	39	68	104	25	87	30
		Mean(SD)	94.9(8.3)	95.1(8.2)	97.0(10.7)	95.5(7.9)	94.3(7.3)	95.0(9.0)	94.8(7.9)	97.1(9.6)
		Median	90.0	91.0	94.0	92.5	90.0	90.0	92.0	98.0
		Min, Max	81,120	82,135	82,130	83,120	81,120	82,120	82,120	83,120
	Visit 2 DBP	Mean(SD)	84.9(9.6)	83.0(7.7)	84.1(8.4)	84.0(9.9)	82.9(8.9)	82.7(9.6)	83.1(8.7)	86.8(12.1)
		Median	83.0	80.0	81.0	80.5	80.0	80.0	80.0	86.5
		Min, Max	60,140	68,103	60,104	65,115	68,130	62,110	60,110	69,120
	Change from Baseline (DBP)	Mean(SD)	-10.1(10.9)	-12.1(9.7)	-12.9(11.1)	-11.4(10.7)	-11.3(10.0)	-12.3(9.5)	-11.8(10.3)	-10.2(10.8)
		Median	-10.0	-10.0	-10.0	-10.0	-10.0	-10.0	-10.0	-10.0
		P value	<0.001	<0.001	<0.001	<0.001	<0.001	<0.001	<0.001	<0.001
CKD	Visit 1 SBP	n	243	65	27	34	98	8	14	7
		Mean(SD)	165.2(18.3)	162.9(16.8)	161.3(21.7)	161.8(18.0)	165.0(18.7)	157.6(10.5)	164.5(18.9)	169.0(15.5)
		Median	166.0	160.0	160.0	160.0	161.5	157.5	160.0	166.0
		Min, Max	122,200	130,200	125,194	135,200	128,200	140,178	140,200	150,197
	Visit 2 SBP	Mean(SD)	145.0(17.7)	142.8(16.9)	140.1(20.2)	142.7(20.0)	147.9(21.5)	134.0(29.7)	146.7(14.2)	139.1(22.9)
		Median	140.0	140.0	140.0	140.0	146.5	127.5	140.0	143.0
		Min, Max	100,200	100,180	100,180	120,200	90,200	94,180	120,170	110,170
	Change from Baseline (SBP)	Mean(SD)	-20.2(22.1)	-20.1(21.5)	-21.1(21.9)	-19.1(18.8)	-17.1(24.7)	-23.6(34.9)	-17.8(22.9)	-29.9(22.0)
		Median	-20.0	-19.0	-20.0	-16.5	-19.0	-35.0	-19.5	-37.0
		P value	<0.001	<0.001	<0.001	<0.001	<0.001	0.0972	0.0123	0.0116
CKD	Visit 1 DBP	n	243	65	27	34	98	8	14	7
		Mean(SD)	96.8(10.2)	94.9(8.6)	92.3(6.9)	96.9(10.4)	93.3(7.6)	98.9(8.7)	94.6(7.5)	100.1(7.8)
		Median	94.0	90.0	90.0	90.0	90.0	98.0	90.0	100.0
		Min, Max	83,144	81,113	83,110	84,120	82,120	90,110	90,110	90,110
	Visit 2 DBP	Mean(SD)	85.3(10.6)	83.4(8.4)	79.0(11.3)	82.8(9.2)	84.0(10.0)	81.5(15.6)	86.8(11.4)	81.7(14.7)
		Median	84.0	80.0	80.0	80.0	80.0	80.0	82.5	75.0
		Min, Max	60,130	68,110	60,100	64,100	60,115	60,100	70,110	65,100
		Median	-10.0	-10.0	-16.0	-10.0	-10.0	-12.5	-10.0	-20.0
		P value	<0.001	<0.001	<0.001	<0.001	<0.001	0.0038	0.0762	0.0109
Dyslipidemia+DM	Visit 1 SBP	n	419	601	87	132	265	81	320	103
		Mean(SD)	159.3(17.5)	160.6(16.2)	159.0(18.6)	163.0(18.0)	160.8(17.2)	162.5(16.6)	162.6(16.4)	168.2(17.1)
		Median	160.0	160.0	160.0	161.5	160.0	160.0	160.0	170.0
		Min, Max	122,200	121,200	125,200	130,200	121,200	127,200	121,200	121,200
	Visit 2 SBP	Mean(SD)	140.1(16.0)	139.4(15.8)	141.3(15.9)	142.5(18.5)	142.1(17.4)	142.4(15.9)	140.8(16.9)	143.0(17.8)
		Median	140.0	140.0	140.0	140.0	140.0	140.0	140.0	140.0
		Min, Max	103,191	70,200	108,180	101,200	90,200	112,180	96,192	110,200
	Change from Baseline (SBP)	Mean(SD)	-19.2(20.2)	-21.2(18.3)	-17.7(19.5)	-20.5(19.3)	-18.8(21.8)	-20.1(22.1)	-21.8(18.9)	-25.1(20.6)
		Median	-20.0	-20.0	-20.0	-20.0	-20.0	-20.0	-20.0	-24.0
		P value	<0.001	<0.001	<0.001	<0.001	<0.001	<0.001	<0.001	<0.001
Dyslipidemia+DM	Visit 1 DBP	n	419	601	87	132	265	81	320	103
		Mean(SD)	92.9(7.3)	94.2(8.0)	94.2(8.5)	93.8(6.9)	93.4(7.9)	94.1(7.0)	94.7(8.3)	96.4(8.6)
		Median	90.0	90.0	90.0	90.0	90.0	90.0	90.0	95.0
		Min, Max	82,124	81,150	81,128	82,120	81,140	83,110	81,138	81,120
	Visit 2 DBP	Mean(SD)	83.3(8.2)	83.0(8.5)	83.8(9.3)	83.9(10.1)	84.0(8.7)	82.7(8.7)	83.6(8.6)	85.2(9.0)
		Median	80.0	80.0	80.0	84.0	82.0	80.0	81.0	82.0
		Min, Max	60,110	60,140	60,106	60,115	60,130	65,120	60,117	60,110
	Change from Baseline (DBP)	Mean(SD)	-9.6(9.9)	-11.2(9.9)	-10.4(11.1)	-9.9(11.0)	-9.4(10.1)	-11.4(9.4)	-11.1(10.1)	-11.2(11.0)
		Median	-10.0	-10.0	-10.0	-10.0	-10.0	-10.0	-10.0	-10.0
		P value	<0.001	<0.001	<0.001	<0.001	<0.001	<0.001	<0.001	<0.001
Dyslipidemia+CAD	Visit 1 SBP	n	150	120	30	54	88	18	69	22
		Mean(SD)	160.9(18.0)	162.1(15.6)	160.6(21.5)	165.3(18.8)	162.8(15.0)	163.5(10.2)	165.7(15.9)	169.0(17.8)
		Median	160.0	160.0	160.0	165.5	160.0	168.0	166.0	172.0
		Min, Max	122,200	126,200	126,200	130,200	128,200	147,180	126,200	126,190
	Visit 2 SBP	Mean(SD)	142.8(16.5)	139.5(16.3)	139.8(18.7)	142.1(16.6)	140.6(17.7)	137.5(14.7)	138.1(17.8)	146.2(16.2)
		Median	140.0	137.0	140.0	140.0	140.0	138.0	138.0	141.5
		Min, Max	110,194	110,190	110,180	110,180	100,200	113,170	96,190	120,190
	Change from Baseline (SBP)	Mean(SD)	-18.2(22.5)	-22.6(18.9)	-20.8(24.8)	-23.2(21.8)	-22.2(21.4)	-26.0(18.1)	-27.6(21.7)	-22.9(24.9)
		Median	-15.0	-20.0	-20.0	-22.5	-20.0	-20.5	-28.0	-25.0
		P value	<0.001	<0.001	<0.001	<0.001	<0.001	<0.001	<0.001	0.0003
Dyslipidemia+CAD	Visit 1 DBP	n	150	120	30	54	88	18	69	22
		Mean(SD)	94.8(8.3)	94.8(8.6)	95.1(8.5)	95.4(7.4)	94.0(7.5)	94.3(9.6)	94.2(7.8)	97.8(10.2)
		Median	90.0	90.5	91.0	92.5	90.0	90.0	90.0	100.0
		Min, Max	82,120	82,135	82,110	83,116	81,120	82,120	82,120	83,120
	Visit 2 DBP	Mean(SD)	85.3(9.8)	82.7(7.7)	83.6(8.0)	84.5(10.0)	82.8(9.1)	80.4(8.4)	81.6(8.5)	87.7(13.1)
		Median	84.0	80.0	80.0	81.5	80.0	80.0	80.0	88.0
		Min, Max	60,140	70,103	60,104	65,115	68,130	62,90	60,110	69,120
	Change from Baseline (DBP)	Mean(SD)	-9.5(11.0)	-12.2(9.6)	-11.5(9.9)	-10.9(11.1)	-11.2(10.5)	-13.9(8.0)	-12.7(10.7)	-10.1(10.6)
		Median	-10.0	-10.0	-10.0	-10.0	-10.0	-14.0	-10.0	-10.0
		P value	<0.001	<0.001	<0.001	<0.001	<0.001	<0.001	<0.001	0.0002
Dyslipidemia+CKD	Visit 1 SBP	n	85	33	15	23	45	3	10	4
		Mean(SD)	165.5(18.2)	161.1(15.4)	165.5(22.6)	164.0(17.9)	161.9(17.0)	157.7(19.1)	165.3(17.5)	166.5(12.5)
		Median	167.0	160.0	160.0	160.0	163.0	155.0	160.0	168.0
		Min, Max	122,200	130,198	126,190	140,200	128,190	140,178	140,200	150,180
	Visit 2 SBP	Mean(SD)	144.7(17.0)	139.9(15.9)	145.8(19.9)	145.0(22.6)	145.8(22.2)	141.7(24.7)	142.4(11.9)	133.5(16.2)
		Median	140.0	140.0	145.0	140.0	142.0	130.0	140.0	135.0
		Min, Max	110,181	100,175	100,180	120,200	90,200	125,170	120,160	114,150
	Change from Baseline (SBP)	Mean(SD)	-20.8(21.3)	-21.2(21.7)	-19.7(26.7)	-19.0(17.5)	-16.1(25.2)	-16.0(40.8)	-22.9(17.1)	-33.0(22.8)
		Median	-20.0	-20.0	-20.0	-17.0	-14.0	-30.0	-20.0	-40.0
		P value	<0.001	<0.001	0.0127	<0.001	<0.001	0.5674	0.0022	0.0631
Dyslipidemia+CKD	Visit 1 DBP	n	85	33	15	23	45	3	10	4
		Mean(SD)	94.9(8.3)	94.7(9.4)	92.6(7.6)	96.5(10.4)	92.5(7.8)	103.3(11.5)	94.4(6.9)	101.0(8.4)
		Median	90.0	90.0	90.0	90.0	90.0	110.0	90.0	102.0
		Min, Max	83,116	81,113	84,110	84,120	82,120	90,110	90,110	90,110
	Visit 2 DBP	Mean(SD)	83.1(9.0)	82.8(8.4)	80.7(13.1)	82.5(9.6)	83.4(9.4)	86.7(18.9)	81.5(5.8)	75.5(10.5)
		Median	80.0	80.0	80.0	80.0	80.0	95.0	80.0	73.5
		Min, Max	67,110	70,110	60,100	69,100	60,103	65,100	70,90	65,90
	Change from Baseline (DBP)	Mean(SD)	-11.8(10.9)	-11.9(10.0)	-11.9(16.9)	-14.0(9.8)	-9.1(10.6)	-16.7(7.6)	-12.9(10.3)	-25.5(11.7)
		Median	-10.0	-11.0	-9.0	-10.0	-8.0	-15.0	-10.0	-27.0
		P value	<0.001	<0.001	0.0164	<0.001	<0.001	0.0634	0.0033	0.0222
DM+CAD	Visit 1 SBP	n	79	75	18	24	47	9	33	13
		Mean(SD)	162.0(18.6)	161.6(15.6)	160.5(21.2)	169.3(17.8)	164.2(15.2)	158.2(8.1)	166.0(16.9)	169.8(17.1)
		Median	162.0	160.0	160.0	169.0	160.0	159.0	165.0	174.0
		Min, Max	122,200	130,200	130,200	138,200	140,200	150,171	136,200	135,190
	Visit 2 SBP	Mean(SD)	142.9(18.8)	141.4(15.7)	144.9(14.1)	146.7(19.4)	143.2(20.4)	131.2(11.4)	144.5(18.1)	143.9(12.4)
		Median	142.0	140.0	143.0	150.0	137.0	136.0	140.0	140.0
		Min, Max	94,192	110,180	120,170	110,180	120,200	113,150	110,190	120,166
	Change from Baseline (SBP)	Mean(SD)	-19.1(23.4)	-20.2(18.8)	-15.6(22.6)	-22.6(21.8)	-21.0(24.4)	-27.0(18.4)	-21.5(19.2)	-25.9(21.9)
		Median	-18.0	-19.0	-17.0	-26.5	-22.0	-20.0	-20.0	-20.0
		P value	<0.001	<0.001	0.0094	<0.001	<0.001	0.0023	<0.001	0.0011
DM+CAD	Visit 1 DBP	n	79	75	18	24	47	9	33	13
		Mean(SD)	93.2(7.5)	95.9(8.8)	95.0(9.8)	96.2(8.3)	93.0(6.9)	90.3(2.9)	93.2(7.2)	94.0(6.3)
		Median	90.0	96.0	91.0	94.0	90.0	90.0	90.0	94.0
		Min, Max	81,118	82,135	82,110	84,116	82,116	87,96	82,110	83,100
	Visit 2 DBP	Mean(SD)	83.9(7.8)	83.4(7.5)	85.3(6.4)	85.8(12.5)	84.2(10.2)	77.3(6.4)	83.4(8.5)	86.4(8.6)
		Median	82.0	80.0	85.0	86.0	80.0	76.0	81.0	88.0
		Min, Max	60,102	70,103	74,99	65,115	70,130	70,90	66,110	69,100
	Change from Baseline (DBP)	Mean(SD)	-9.3(9.0)	-12.5(8.3)	-9.7(8.3)	-10.4(14.9)	-8.7(8.5)	-13.0(5.3)	-9.8(9.7)	-7.6(6.5)
		Median	-10.0	-11.0	-10.0	-10.0	-10.0	-13.0	-10.0	-10.0
		P value	<0.001	<0.001	0.0001	0.0023	<0.001	<0.001	<0.001	0.0011
DM+CKD	Visit 1 SBP	n	72	30	9	17	44	7	12	3
		Mean(SD)	165.2(17.6)	162.5(15.9)	167.4(18.5)	166.2(17.0)	162.5(16.7)	157.3(11.3)	161.9(17.3)	165.3(5.0)
		Median	170.0	168.5	170.0	160.0	160.0	155.0	156.5	166.0
		Min, Max	122,200	130,198	136,190	140,200	128,200	140,178	140,200	160,170
	Visit 2 SBP	Mean(SD)	147.3(18.3)	144.9(16.4)	149.0(12.9)	151.1(22.7)	148.6(22.6)	139.7(26.9)	147.8(15.1)	133.7(23.7)
		Median	146.5	140.0	145.0	147.0	148.0	130.0	145.0	127.0
		Min, Max	110,196	108,180	134,170	120,200	90,200	107,180	120,170	114,160
	Change from Baseline (SBP)	Mean(SD)	-17.9(22.2)	-17.6(20.7)	-18.4(26.2)	-15.1(13.1)	-13.8(25.8)	-17.6(32.9)	-14.1(21.1)	-31.7(27.8)
		Median	-20.0	-20.0	-20.0	-15.0	-10.0	-30.0	-14.5	-43.0
		P value	<0.001	<0.001	0.0679	0.0002	0.0009	0.2069	0.0409	0.1871
DM+CKD	Visit 1 DBP	n	72	30	9	17	44	7	12	3
		Mean(SD)	93.5(7.8)	94.1(8.5)	91.6(6.0)	95.0(9.9)	90.5(6.1)	98.7(9.4)	94.5(7.8)	98.0(7.2)
		Median	90.0	90.0	90.0	90.0	90.0	96.0	90.0	100.0
		Min, Max	83,115	82,110	84,100	86,120	82,110	90,110	90,110	90,104
	Visit 2 DBP	Mean(SD)	83.8(8.5)	82.1(8.3)	82.1(12.4)	84.1(8.6)	82.3(9.2)	84.6(14.0)	87.5(12.2)	80.0(18.0)
		Median	80.5	80.0	80.0	80.0	80.0	80.0	85.0	75.0
		Min, Max	69,110	69,100	65,100	69,100	60,100	65,100	70,110	65,100
	Change from Baseline (DBP)	Mean(SD)	-9.8(9.6)	-12.0(11.6)	-9.4(16.1)	-10.9(8.3)	-8.3(10.3)	-14.1(7.6)	-7.0(16.3)	-18.0(15.7)
		Median	-10.0	-10.0	-4.0	-10.0	-8.0	-10.0	-10.0	-25.0
		P value	<0.001	<0.001	0.1159	<0.001	<0.001	0.0027	0.1640	0.1857
CKD+CAD	Visit 1 SBP	n	22	7	2	7	10	1	3	1
		Mean(SD)	160.8(17.9)	154.6(13.2)	131.0(7.1)	167.6(18.8)	165.9(11.9)	160.0(.)	186.7(11.5)	180.0(.)
		Median	160.0	150.0	131.0	180.0	170.0	160.0	180.0	180.0
		Min, Max	122,190	140,171	126,136	140,187	150,190	160,160	180,200	180,180
	Visit 2 SBP	Mean(SD)	141.5(18.3)	140.9(21.3)	146.5(33.2)	146.4(18.8)	145.7(30.5)	120.0(.)	156.7(5.8)	143.0(.)
		Median	140.0	130.0	146.5	140.0	145.0	120.0	160.0	143.0
		Min, Max	120,172	120,180	123,170	120,178	100,200	120,120	150,160	143,143
	Change from Baseline (SBP)	Mean(SD)	-19.3(24.2)	-13.7(28.8)	15.5(26.2)	-21.1(23.9)	-20.2(33.6)	-40.0(.)	-30.0(17.3)	-37.0(.)
		Median	-20.0	-20.0	15.5	-11.0	-25.0	-40.0	-20.0	-37.0
		P value	0.0012	0.2552	0.5560	0.0576	0.0899		0.0955	
CKD+CAD	Visit 1 DBP	n	22	7	2	7	10	1	3	1
		Mean(SD)	94.7(7.3)	91.7(8.5)	94.5(14.8)	98.1(10.9)	90.1(4.0)	90.0(.)	96.7(11.5)	110.0(.)
		Median	90.0	90.0	94.5	91.0	90.0	90.0	90.0	110.0
		Min, Max	86,110	83,110	84,105	90,116	84,100	90,90	90,110	110,110
	Visit 2 DBP	Mean(SD)	81.7(7.6)	84.3(9.8)	82.5(3.5)	81.0(3.7)	80.8(7.5)	80.0(.)	86.7(5.8)	72.0(.)
		Median	80.0	80.0	82.5	80.0	80.0	80.0	90.0	72.0
		Min, Max	70,100	70,100	80,85	77,89	68,90	80,80	80,90	72,72
	Change from Baseline (DBP)	Mean(SD)	-13.0(9.7)	-7.4(10.9)	-12.0(11.3)	-17.1(8.6)	-9.3(10.2)	-10.0(.)	-10.0(10.0)	-38.0(.)
		Median	-10.0	-8.0	-12.0	-14.0	-9.0	-10.0	-10.0	-38.0
		P value	<0.001	0.1203	0.3743	0.0019	0.0184		0.2254	
Dyslipidemia+DM+CAD	Visit 1 SBP	n	66	57	16	21	43	7	29	11
		Mean(SD)	160.9(17.9)	162.1(13.6)	161.2(22.4)	172.9(15.9)	163.2(14.9)	158.9(8.9)	166.5(15.6)	168.7(18.2)
		Median	162.0	162.0	160.0	170.0	160.0	159.0	168.0	174.0
		Min, Max	122,200	130,190	130,200	140,200	140,200	150,171	139,200	135,190
	Visit 2 SBP	Mean(SD)	142.8(17.6)	139.9(15.6)	145.2(14.9)	150.5(17.4)	142.6(20.0)	132.1(12.1)	144.4(19.3)	141.4(11.2)
		Median	141.0	137.0	143.5	150.0	137.0	136.0	140.0	140.0
		Min, Max	110,180	110,180	120,170	110,180	120,200	113,150	110,190	120,160
	Change from Baseline (SBP)	Mean(SD)	-18.1(24.0)	-22.2(16.6)	-16.0(24.0)	-22.4(23.3)	-20.6(24.5)	-26.7(20.1)	-22.0(18.8)	-27.4(23.6)
		Median	-13.0	-20.0	-20.0	-26.0	-22.0	-20.0	-20.0	-30.0
		P value	<0.001	<0.001	0.0175	0.0003	<0.001	0.0126	<0.001	0.0032
Dyslipidemia+DM+CAD	Visit 1 DBP	n	66	57	16	21	43	7	29	11
		Mean(SD)	93.1(6.9)	95.5(9.0)	93.8(9.5)	97.6(7.8)	92.7(7.0)	89.6(2.3)	92.6(6.7)	94.0(6.3)
		Median	90.0	96.0	90.0	96.0	90.0	90.0	90.0	94.0
		Min, Max	82,110	83,135	82,110	90,116	82,116	87,94	82,110	83,100
	Visit 2 DBP	Mean(SD)	83.8(7.8)	82.9(7.8)	84.9(6.7)	87.0(12.8)	84.1(10.5)	75.1(4.6)	83.1(8.9)	85.9(9.3)
		Median	82.5	80.0	82.0	89.0	80.0	75.0	80.0	86.0
		Min, Max	60,100	70,103	74,99	65,115	70,130	70,82	66,110	69,100
	Change from Baseline (DBP)	Mean(SD)	-9.3(9.0)	-12.6(8.5)	-8.8(8.3)	-10.6(15.6)	-8.5(8.6)	-14.4(5.0)	-9.5(10.1)	-8.1(6.6)
		Median	-10.0	-11.0	-9.0	-10.0	-10.0	-16.0	-10.0	-10.0
		P value	<0.001	<0.001	0.0007	0.0054	<0.001	0.0003	<0.001	0.0023
Dyslipidemia+DM+CKD	Visit 1 SBP	n	38	18	8	13	27	3	8	2
		Mean(SD)	164.7(18.7)	161.4(16.6)	166.6(19.6)	170.4(16.8)	158.1(14.6)	157.7(19.1)	161.6(14.3)	168.0(2.8)
		Median	170.0	166.0	165.0	166.0	157.0	155.0	160.0	168.0
		Min, Max	122,200	130,198	136,190	146,200	128,190	140,178	140,180	166,170
	Visit 2 SBP	Mean(SD)	144.0(16.0)	143.4(13.5)	150.9(12.4)	157.2(22.1)	146.6(23.4)	141.7(24.7)	143.0(13.4)	120.5(9.2)
		Median	141.0	140.0	147.5	160.0	140.0	130.0	140.0	120.5
		Min, Max	120,180	125,175	140,170	122,200	90,200	125,170	120,160	114,127
	Change from Baseline (SBP)	Mean(SD)	-20.7(23.0)	-18.0(18.9)	-15.8(26.7)	-13.2(13.0)	-11.5(25.8)	-16.0(40.8)	-18.6(12.5)	-47.5(6.4)
		Median	-20.0	-20.0	-15.0	-10.0	-10.0	-30.0	-19.5	-47.5
		P value	<0.001	0.0008	0.1388	0.0034	0.0292	0.5674	0.0039	0.0601
Dyslipidemia+DM+CKD	Visit 1 DBP	n	38	18	8	13	27	3	8	2
		Mean(SD)	93.2(8.2)	93.2(8.7)	91.0(6.1)	96.5(11.0)	89.0(4.5)	103.3(11.5)	94.3(7.3)	97.0(9.9)
		Median	90.0	90.0	90.0	91.0	90.0	110.0	90.0	97.0
		Min, Max	83,110	83,110	84,100	86,120	82,105	90,110	90,110	90,104
	Visit 2 DBP	Mean(SD)	82.7(8.1)	81.4(7.3)	82.4(13.2)	84.6(9.6)	81.4(8.9)	86.7(18.9)	81.3(6.4)	70.0(7.1)
		Median	80.0	80.0	80.0	80.0	80.0	95.0	80.0	70.0
		Min, Max	69,110	70,100	65,100	69,100	60,100	65,100	70,90	65,75
	Change from Baseline (DBP)	Mean(SD)	-10.5(10.4)	-11.7(11.5)	-8.6(17.0)	-11.9(9.0)	-7.7(10.3)	-16.7(7.6)	-13.0(11.6)	-27.0(2.8)
		Median	-10.0	-13.5	-4.0	-10.0	-7.0	-15.0	-10.0	-27.0
		P value	<0.001	0.0005	0.1939	0.0004	0.0007	0.0634	0.0158	0.0471
Dyslipidemia+CAD+CKD	Visit 1 SBP	n	17	5	2	5	8		2	1
		Mean(SD)	159.4(18.0)	160.4(10.5)	131.0(7.1)	170.6(17.1)	162.4(9.1)		180.0(0.0)	180.0(.)
		Median	160.0	162.0	131.0	180.0	166.5		180.0	180.0
		Min, Max	122,190	149,171	126,136	146,187	150,170		180,180	180,180
	Visit 2 SBP	Mean(SD)	141.1(19.5)	137.2(13.0)	146.5(33.2)	149.0(22.4)	139.6(31.4)		160.0(0.0)	143.0(.)
		Median	140.0	130.0	146.5	152.0	132.0		160.0	143.0
		Min, Max	120,172	130,160	123,170	120,178	100,200		160,160	143,143
	Change from Baseline (SBP)	Mean(SD)	-18.4(25.6)	-23.2(20.1)	15.5(26.2)	-21.6(25.6)	-22.8(37.2)		-20.0(0.0)	-37.0(.)
		Median	-20.0	-32.0	15.5	-11.0	-30.0		-20.0	-37.0
		P value	0.0093	0.0614	0.5560	0.1320	0.1277		<0.001	
Dyslipidemia+CAD+CKD	Visit 1 DBP	n	17	5	2	5	8		2	1
		Mean(SD)	94.5(7.9)	92.4(10.3)	94.5(14.8)	95.4(11.5)	90.1(4.5)		90.0(0.0)	110.0(.)
		Median	90.0	90.0	94.5	90.0	90.0		90.0	110.0
		Min, Max	86,110	83,110	84,105	90,116	84,100		90,90	110,110
	Visit 2 DBP	Mean(SD)	82.6(7.6)	86.0(8.9)	82.5(3.5)	81.4(4.5)	79.8(7.7)		85.0(7.1)	72.0(.)
		Median	80.0	80.0	82.5	80.0	80.0		85.0	72.0
		Min, Max	70,100	80,100	80,85	77,89	68,90		80,90	72,72
	Change from Baseline (DBP)	Mean(SD)	-11.9(9.0)	-6.4(11.1)	-12.0(11.3)	-14.0(7.5)	-10.4(11.0)		-5.0(7.1)	-38.0(.)
		Median	-10.0	-8.0	-12.0	-10.0	-9.0		-5.0	-38.0
		P value	<0.001	0.2653	0.3743	0.0141	0.0322		0.5000	
DM+CAD+CKD	Visit 1 SBP	n	9	5	1	3	7	1	3	
		Mean(SD)	160.1(23.6)	150.0(12.7)	136.0(.)	175.7(14.0)	161.3(9.3)	160.0(.)	186.7(11.5)	
		Median	170.0	149.0	136.0	180.0	163.0	160.0	180.0	
		Min, Max	122,189	140,171	136,136	160,187	150,170	160,160	180,200	
	Visit 2 SBP	Mean(SD)	144.8(21.3)	145.2(24.4)	170.0(.)	163.3(13.3)	149.6(30.4)	120.0(.)	156.7(5.8)	
		Median	147.0	136.0	170.0	160.0	150.0	120.0	160.0	
		Min, Max	120,172	120,180	170,170	152,178	120,200	120,120	150,160	
	Change from Baseline (SBP)	Mean(SD)	-15.3(32.3)	-4.8(29.9)	34.0(.)	-12.3(19.7)	-11.7(34.4)	-40.0(.)	-30.0(17.3)	
		Median	-10.0	-19.0	34.0	-2.0	-6.0	-40.0	-20.0	
		P value	0.1918	0.7376		0.3907	0.4021		0.0955	
DM+CAD+CKD	Visit 1 DBP	n	9	5	1	3	7	1	3	
		Mean(SD)	91.4(5.1)	88.8(3.3)	84.0(.)	99.0(14.7)	88.7(2.2)	90.0(.)	96.7(11.5)	
		Median	90.0	90.0	84.0	91.0	90.0	90.0	90.0	
		Min, Max	86,100	83,91	84,84	90,116	84,90	90,90	90,110	
	Visit 2 DB	Mean(SD)	80.9(5.3)	84.0(11.4)	80.0(.)	82.0(6.2)	82.9(7.2)	80.0(.)	86.7(5.8)	
		Median	80.0	80.0	80.0	80.0	82.0	80.0	90.0	
		Min, Max	70,90	70,100	80,80	77,89	70,90	80,80	80,90	
	Change from Baseline (DBP)	Mean(SD)	-10.6(7.9)	-4.8(11.3)	-4.0(.)	-17.0(8.9)	-5.9(7.6)	-10.0(.)	-10.0(10.0)	
		Median	-10.0	-3.0	-4.0	-14.0	-4.0	-10.0	-10.0	
		P value	0.0039	0.3977		0.0803	0.0870		0.2254	
